# DNA Damage Repair Defects and Targeted Radionuclide Therapies for Prostate Cancer: Does Mutation Really Matter? A Systematic Review

**DOI:** 10.3390/life13010055

**Published:** 2022-12-24

**Authors:** Luca Filippi, Barbara Palumbo, Oreste Bagni, Viviana Frantellizzi, Giuseppe De Vincentis, Orazio Schillaci

**Affiliations:** 1 Nuclear Medicine Unit, “Santa Maria Goretti” Hospital, Via Antonio Canova, 04100 Latina, Italy; 2 Section of Nuclear Medicine and Health Physics, Department of Medicine and Surgery, Università Degli Studi di Perugia, Piazza Lucio Severi 1, 06132 Perugia, Italy; 3 Department of Radiological Sciences, Oncology and Anatomo-Pathology, Sapienza, University of Rome, 00185 Rome, Italy; 4 Department of Biomedicine and Prevention, University Tor Vergata, Viale Oxford 81, 00133 Rome, Italy

**Keywords:** prostate cancer, radium, PSMA, precision oncology, radionuclide therapy

## Abstract

The aim of the present review was to assess the impact of DNA damage repair (DDR) mutations on response and outcome of patients (pts) affected by advanced prostate cancer (PCa) submitted to radionuclide therapies with [^223^Ra]RaCl_2_ (^223^Ra-therapy) or prostate specific membrane antigen (PSMA) ligands. A systematic literature search according to PRISMA criteria was made by using two main databases. Only studies published up until to October 2022 in the English language with ≥10 enrolled patients were selected. Seven studies including 326 pts, of whom 201 (61.6%) harboring DDR defects, were selected. The majority of selected papers were retrospective and four out of seven (57.1%) had small sample size (<50 pts). Three out of seven (42.8%) studies reported a more favorable outcome (overall or progression free survival) after therapy with alpha emitters (^223^Ra-therapy or [^225^Ac]Ac-PSMA-617) in subjects with DDR defects with respect to those without mutations. In two studies employing alpha or beta emitters ([^177^Lu]/[^225^Ac]-PMSA), no significant benefit was registered in pts harboring DDR defects. In all but one paper, no significant difference in response rate was reported among pts with or without DDR mutations. Although preliminary and biased by the retrospective design, preliminary data suggest a trend towards a longer survival in PCa pts harboring DDR defects submitted to radionuclide targeted therapy with alpha emitters.

## 1. Introduction

Prostate cancer (PCa) is the second most commonly diagnosed malignancy in men and a cancer-related leading cause of death worldwide [1]. Localized PCa is commonly treated with surgery or radiation therapy (RT) with radical intent, while the standard of care for advanced disease is represented by androgen deprivation therapy (ADT) [2]. However, following a variable period of hormonal therapy, the majority of PCa patients evolve towards a hormone-refractory disease, namely castration-resistant prostate cancer (CRPC). The clinical spectrum of CRPC might range from a condition of limited tumor burden with few and mild symptoms to a more severe and deeply debilitating state, with diffuse metastatization (mCRPC) and poor prognosis [3].

In recent years, aside from taxane-based chemotherapy, novel therapeutic options have been introduced for the management of mCRPC, such as androgen-receptor signaling inhibitors (ARSI), including abiraterone, enzalutamide, darolutamide and apalutamide, and immunotherapy with sipuleucel-T or the immune checkpoint blocker pembrolizumab [4].

In this scenario, radionuclide-based therapies have been implemented for the management of mCRPC. Following the results of the ALSYMPCA trial [5], which demonstrated a survival benefit in mCRPC patients treated with [^223^Ra]RaCl_2_ (^223^Ra-therapy) with respect to those submitted to the best standard of care, the U.S. Food and Drug Administration (FDA) approved Xofigo^TM^ (Bayer HealthCare Pharmaceuticals Inc.), for the targeted alpha therapy (TAT) of mCRPC [6]. Since then, TAT with ^223^Ra-therapy has been widely applied in clinical practice, resulting impactful on patients’ survival and quality of life [7,8]. Nevertheless, ^223^Ra-therapy is a bone-seeking agent; therefore, its therapeutic effect is limited only to bone metastases. Other therapeutic options are needed to manage mCRPC subjects bearing both skeletal and visceral localizations. 

In recent years, prostate cancer membrane antigen (PSMA), a type II integral membrane glycoprotein that is strongly over-expressed in PCa and minimally detectable in prostate normal tissue, has emerged as an attractive biomarker in the field. Some small molecules exhibiting inhibitory activity towards the PSMA-enzymatic domain have been synthesized and labeled with radionuclides suitable for imaging (photon- or positron-emitters) or therapy (beta or alpha-particles’ emitters), with the aim of combining diagnosis and therapy in a unique approach, namely “theranostics” [9,10,11,12]. 

International guidelines recommend positron emission computed tomography (PET/CT) with PSMA-ligands (e.g., [^68^Ga]Ga-PSMA-11), as the gold standard for the staging of high-risk PCa and for the diagnosis of biochemical recurrence (BCR). On the therapeutic side, the international, open-label, phase 3 VISION proved that mCRPC subjects treated with [^177^Lu]Lu-PSMA-617 had both longer imaging-based progression-free survival and overall survival with respect to subjects submitted to the best standard of care [13]. As a result, [^177^Lu]Lu-PSMA-617 (Pluvicto^TM^) has been recently FDA-approved for the radioligand therapy (RLT) of mCRPC, previously tested for PSMA-expression by PSMA PET/CT [14]. 

DNA damage repair (DDR) genes present a crucial role to preserve human genome integrity, by detecting eventual DNA damage and triggering all the mechanisms involved in DNA repair [15]. Mutations in DDR genes, frequently found in advanced PCa, have been identified as valuable biomarkers for patients’ selection before targeted therapies with poly-ADP ribose polymerase (PARP) inhibitors [16]. As a matter of fact, the so-called “synthetic lethality” phenomenon occurs when two causes, each of whom would be unable to independently cause cell death, are combined to determine a lethal damage. In this regard, PARP-inhibitors have been found to determine synthetic lethality in PCa harboring DDR genes’ mutations [17].

Both ^223^Ra-therapy and RLT, although working with different approaches, have DNA as their main target to determine radiation-induced biological effects. Worthy of note, the distribution and type of DNA damage mainly depends on the nature and energy of the emitted particles. In light of the above, it has been hypothesized that mutations in DDR genes might influence PCa sensitivity to DNA-damage induced by radionuclide-based therapy. However, to the best of our knowledge, the scientific data on this topic are still limited.

Therefore, the aim of the present systematic review was to provide a comprehensive overview of the existing scientific literature on the role of DDR mutations in PCa patients’ response to targeted radionuclide therapies, with the aim to give an answer to the following question: “Does mutation really matter?”

## 2. Materials and Methods

### 2.1. Search Strategy

An electron search up until October 2022 in PubMed and Scopus databases was carried out according to the Preferred Reporting Items for Systematic reviews and Meta-analyses (PRISMA) guidelines for retrieving all the clinical studies concerning the impact of DDR genes’ defect and targeted radionuclide therapy [18]. 

The search strategy was built using the terms: (A) “DNA damage gene” OR “Homologous Repair Gene Defects” OR “DNA repair” OR “DNA mutations” AND (B) “Radium-223” OR “Radioligand Therapy” OR “PSMA”. The following types of studies were considered: cohort studies, clinical trials, prospective studies, and retrospective cohorts. Case reports, conference proceedings, editorial commentaries, interesting images, studies on healthy volunteers, and letters to the editor were excluded. Only studies published up from January 2012 to October 2022, limited to humans, in the English language and with a cohort of ≥10 patients were selected.

Two reviewers (V.F., L.F.) conducted the literature search and independently appraised each article using a standard protocol and data extraction. The reference lists of the selected studies were carefully checked to identify any additional relevant literature. 

From each study extracted data were: type of the study (prospective, retrospective, etc.), year and location of the study, sample size, employed radiopharmaceuticals, response rate, and follow-up data. Studies with incomplete technical or clinical data were considered ineligible. Any discrepancy was resolved by discussion among authors. 

### 2.2. Quality of the Selected Studies 

Selected imaging studies were analyzed using a modified version of the Critical Appraisal Skills Programme (CASP) (https://casp-uk.net/aboutus, accessed on 24 November 2022) checklist for Cohort Study. Critical appraisal was performed by two reviewers (L.F. and V.F.), and discrepancies, if any, were resolved by discussion among researchers.

## 3. Results

### 3.1. Analysis of the Evidence

**Figure 1:**
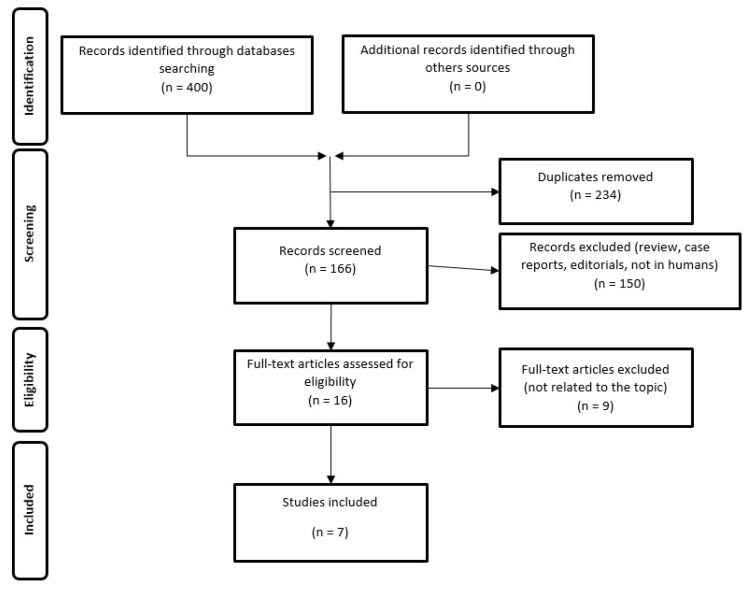
Schematic representation of PRISMA workflow for manuscripts’ selection.

The resulting PRISMA search strategy is shown in Figure 1. From the systematic literature search, seven papers were selected, for an overall number of 326 enrolled mCRPC patients, of whom 201 (61.6%) harboring DDR defects, submitted to targeted radionuclide therapy. Table 1 summarizes the main findings of the selected manuscripts.

From the analysis of the selected papers, we identified two main thematic areas: (1) targeted alpha therapy with ^223^Ra-therapy (papers = 3, 42.8%) (2) PSMA-targeted radionuclide therapy, employing alpha-emitters ([^225^Ac]Ac-PSMA-617, n = 2, 28.6%) or both alpha and beta emitters ([^225^Ac/^177^Lu]-PSMA, n = 2, 28.6%). 

In case of ^223^Ra-therapy, response was mainly assessed on the basis of decrease in alkaline phosphatase (ALP) according to the Prostate Cancer Working Group 3 (PCWG3) criteria, evaluated at 6–12 weeks post therapy, with a reported response rate resulting in 33–69% [26]. In case of PSMA-directed RLT, response was defined on the basis of decline in prostate specific antigen (PSA) at 6 weeks after therapy, with a response rate range of 26.7–69%. Imaging response was determined, according to morphological or functional criteria only in a minority of patients. 

**Figure 2:**
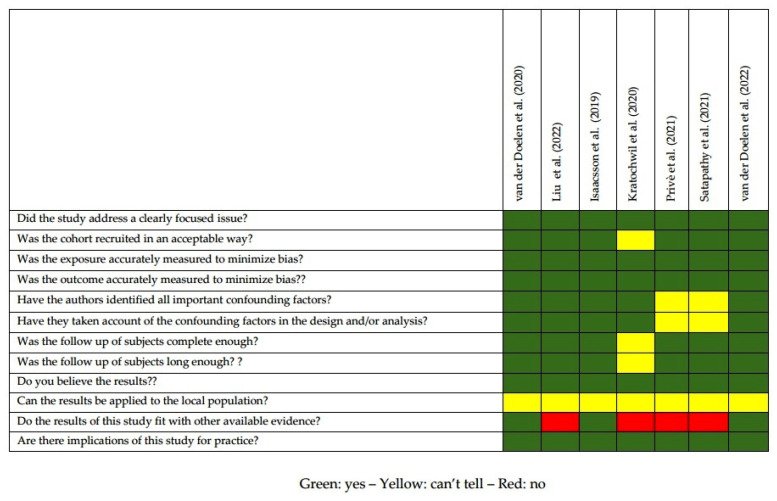
Quality appraisal of selected articles using CASP checklist for cohort studies [19,20,21,22,23,24,25].

The quality appraisal of the selected studies is represented in Figure 2. The majority of the selected papers (71.4%) were retrospective, while two were observational cohort studies. Since both ^223^Ra-therapy and RLT have been recently implemented, all the studies were carried out in a relatively narrow range of time (2019–2022). A major limitation of the various studies was represented by the limited number of included patients (range 10–127): four papers (57.1%) had a cohort <50 subjects. Six studies tested a panel of various DDR genes, while 1 paper was focused only on genes implicated in the homologous recombination (HR) pathway. In the majority of cases (six out seven papers, 85.7%), final outcome (overall survival/OS or progression free survival/PFS) was set as the study endpoint, while one paper was aimed to define the prevalence of DDR mutations in mCRPC refractory to [^225^Ac]Ac-PSMA-617. 

The findings of the selected papers for each thematic area are described in the following paragraphs.

#### 3.1.1. Targeted Alpha Therapy with ^223^Ra-Therapy

Isaacsson and coworkers focused their retrospective study on mutations in DDR genes involved in the HR pathway, a mechanism particularly relevant in case of radiation-induced double-stranded DNA (ds-DNA) breaks [19]. The authors enrolled 28 men with bone metastases from mCRPC, tested by next generation sequencing (NGS) for HR mutations, submitted to ^223^Ra-therapy. HR mutations were identified in 10 cases (HR+) while 18 patients did not show any abnormality (HR-). The primary endpoint was to assess whether HR+ patients had a higher clinical benefit from ^223^Ra-therapy with respect to HR- subjects. Among all the enrolled men, 64% had ALP response within 12 weeks, assessed following PCWG3 criteria, with a significantly higher rate of response in HR+ than in HR- men (80% vs. 39%, respectively). In addition, HR+ patients exhibited significantly longer time to ALP progression and prolonged time to next systemic therapy. Notably, HR+ positive patients had a meaningfully more favorable (although not significantly) outcome than HR- ones, with an overall survival (OS) of 36.9 vs. 19.0 months (95% confidence interval/CI = 0.7–15.6).

In a bicentric retrospective study carried out by van der Doelen et al., ninety-three mCRPC patients, without evidence of visceral metastases, were screened for germline/somatic DDR mutations by NGS before ^223^Ra-therapy [20]. Among the enrolled subjects, 28 cases (30.1%) harbored DDR aberrations and were classified as DDR+, while the remaining patients were categorized as DDR-. The most frequently mutated genes were ataxia-telangiectasia mutated (ATM, 8.6%), breast cancer gene 2 (BRCA2, 7.5%) and cyclin-dependent kinase 12 (CDK-12, 4.3%). The study primary endpoint was OS: DDR+ patients had a significantly longer median OS than DDR- ones (median 36.3 vs. 17.0 months; 95% CI% 1.21–4.32). In addition, the authors analyzed 2 secondary endpoints: time to ALP progression (TAP) and time to subsequent treatment (TST), that also resulted longer in DDR+ cohort than in DDR- one. Notably, biochemical PSA and ALP response did not meaningfully differ among groups, with a 61.3% of patients showing a significant ALP response. Worthy of note, DDR+ patients received a higher number of sequential therapies, including PARP-inhibitors. 

In a recently published paper, Liu and coworkers retrospectively evaluated the response to ^223^Ra-therapy (primary endpoint) and survival (both OS and PFS) in 127 mCRPC bearing a huge range of DDR defects [23]. In this cohort, the most frequently mutated genes were transformation-related protein 53 (TP53, 51.7%), BRCA 1/2 (15%), and phosphatase and tensin homolog (PTEN, 13.4%). In the entire cohort, 22.6% of patients exhibited PSA response, while 69.8% were ALP-responders. As far as it concerns the primary endpoint, none among the various analyzed DDR mutations resulted a relevant predictor of PSA or ALP response. Similarly, no significant impact of DDR aberrations was registered on OS and PFS, although TMPRSS2-ERG mutation was associated with a lower OS (15.4 mo), while RB deletion with a shorter PFS (6 mo).

#### 3.1.2. PSMA-Targeted Alpha and Beta Radioligand Therapy 

The prevalence of DDR defects was retrospectively assessed by Kratochwil and coworkers in mCRPC patients with visceral and skeletal metastases submitted to TAT with [^225^Ac]Ac-PSMA-617 [21]. From an initial cohort of 60 subjects, the authors identified 10 patients with poor response to RLT in spite of sufficiently high and homogeneous PSMA-expression at tumor sites. The selected patients were then screened for lesions suitable for biopsy in order to stratify subjects for therapy with olaparib (in case of DDR mutations) or platin chemotherapy (in case of neuroendocrine differentiation): biopsy was carried out in 7 out 10 cases. A huge panel including 37 genes involved in DDR was tested by NGS: the most frequently found mutations were TP53, Checkpoint Kinase 2 (CHEK2) and ATM, with an average number of deleterious or presumably deleterious mutations of 2.2 (range, 0–6) per patient. Therefore, the authors concluded that DDR abnormalities might represent a frequent occurrence in mCRPC patients refractory to [^225^Ac]Ac-PSMA-617. Nevertheless, the results obtained in “poorly responders” were not compared with those detectable in good-responders. It is worth mentioning that all the enrolled patients were heavily pre-treated: in particular, 4 had been previously treated with the beta-emitter [^177^Lu]Lu-PSMA-617, with consequent potentially relevant pre-mutagenic impact. 

In an observational cohort study carried out by Privè and colleagues, the response to PSMA-RLT with beta or alpha emitters ([^177^Lu/^225^Ac]-PSMA-617 or PSMA-I&T) was assessed through a minimum 6 weeks’ follow-up in an overall number of 40 mCRPC patients [22]. Seventeen of 40 subjects harbored mutations and were classified as DDR+, being BRCA1/2 the most commonly registered mutated genes. All participants were tested for PSMA-expression by PET/CT with [^68^Ga] or [^18^F]-PSMA and received [^177^Lu] or [^225^Ac]-RLT according to radiopharmaceuticals’ availability or physicians’ preference. No significant differences among the two cohorts were found in PSA response (59% in DDR+ vs. 65% in DDR-) or in term of PFS (5.9 vs. 6.4 months in DDR+ and DDR-, respectively; 95% CI 0.58–2.25) and OS (11.1 vs. 10.7 months in DDR+ and DDR-, respectively; 95% CI: 0.68–2.91). 

In a retrospective assessment of a prospectively maintained registry, Satapathy and coworkers analyzed the prevalence and clinical impact of DDR mutations on outcome in mCRPC patients submitted to RLT [25]. The authors identified 15 patients, of whom 12 submitted to [^177^Lu]Lu-PSMA-617and 3 treated with [^225^Ac]Ac-PSMA-617, investigated for DDR mutations by NGS before treatment. In 10 out 15 cases, DDR alterations were found, being ATM, BRCA2, and TP53 the most commonly mutated genes. In all cases, subjects were selected for RLT on the basis of [^68^Ga]Ga-PSMA-11 PET/CT’s results. After RLT, all subjects were followed-up every three weeks and response was assessed by PET/CT examination after 6 weeks. The primary endpoint of the study was PFS, in addition biochemical response according to PCWG3 and radiological response according to Response Evaluation Criteria in Solid Tumors (RECIST 1.1) were determined [27]. As far as it concerns the primary endpoint, the entire cohort had a median PFS of 3 months, while median OS resulted in 9 months, with no significant differences among patients with or without DDR alterations. A PSA response was registered in the 26.7% of cases, while radiological response was evaluated only in 10/15 cases with an overall response rate of 12.5%, without relevant difference among subjects with or without DDR mutations. In addition, in univariate analysis, DDR aberration did not represent a significant predictor of response and outcome. 

Van der Doelen and coworkers have recently carried out an observational cohort study including 13 consecutive mCRPC patients (of whom, 2 previously treated with [^177^Lu]Lu-PSMA-617), submitted to [^225^Ac]Ac-PSMA-617 RLT: in all cases PSMA-expression was tested with [^68^Ga]Ga-PSMA-11 PET/CT and on tumor tissue by histochemical analysis before therapy enrollment [24]. Primary endpoint of the study was OS, while secondary endpoints were clinical response (time between RLT start and no longer benefit), biochemical response as defined by PCWG3 and radiological response assessed according to both RECIST and PET response criteria in solid tumors (PERCIST) [28]. The median OS resulted in 8.5 months in the entire cohort. By Kaplan–Meier analysis, a trend towards a longer survival was associated with the following prognostic factors: lack of previous [^177^Lu]Lu-PSMA-617 therapy, high PSMA density at histochemical analysis, and the presence of DDR defects. 

## 4. Discussion

In recent years, we have been witnessing an impressive development in the field of targeted radionuclide therapy, firstly with the approval of ^223^Ra-therapy for PCa therapy, then followed by the authorization of [^177^Lu]Lu-oxodotreotide for the management of neuroendocrine tumors (NET) and, even more recently, by the implementation of [^177^Lu]Lu-PSMA-617 for mCRPC [14,29,30,31]. 

**Figure 3:**
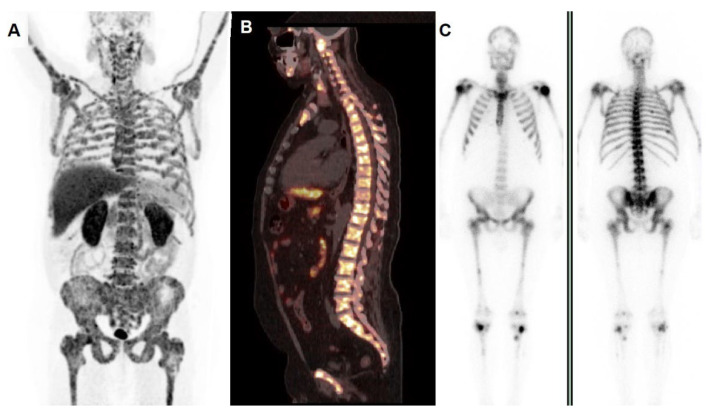
A 57-year-old man, diagnosed with locally advanced PCa in 2019 (Gleason score 4 + 5, ISUP 5), progressive after a sequence of various therapeutic regimens due to skeletal metastases (ADT, second generation androgens, taxane chemotherapy), positive for ATM germline mutation at NGS analysis. He was referred to our facility for ^223^Ra-therapy: pre-therapy whole body PET/CT with ^18^F-choline (**A**) showed increased tracer incorporation in the entire skeleton, as well depicted also by sagittal fused PET/CT of the spine (**B**). PET/CT’s findings were consistent with bone scan (**C**), left side—anterior view, right side—posterior view) demonstrating a typical pattern of “super bone”. After 2 cycles, a biochemical ALP response was registered (79 U/L post 2 cycles vs. 326 U/L at baseline). Unfortunately, the patient discontinued therapy due overlapping pulmonary complications.

The resurgence of interest for radionuclide-based treatments in oncology, on the one hand, has meaningfully impacted on the therapeutic landscape of several oncological conditions and, on the other hand, has generated an unmet need for patients’ stratification in order to identify subjects who are more likely to respond to a certain therapeutic regimen. In this regard, it has been hypothesized that DDR defects might be applied as biomarkers for selection before enrollment for radionuclide therapies [32]. This assumption was mainly based on the following issues: (1) PCa tumors with DDR mutations were found to be characterized by higher Gleason scores and PSMA expression; (2) the association of DDR defects with radiation-induced DNA-damage was thought to be involved in the mechanism of “synthetic lethality” [32,33]. An example of a mCRPC patient harboring a DDR defect, selected among our series, is depicted in Figure 3.

From the careful analysis of the selected papers, some considerations have to be made about the role of DDR alterations in the clinical setting of PCa-targeted radionuclide therapy.

All the selected papers show that the prevalence of DDR defects is relatively high in patients with advanced PCa, submitted to systemic therapies, in agreement with previously published data [34]. In addition, most of the analyzed papers (5 out of 7, 71.4%) did not show any significant difference in response rate between patients with and without DDR defects. By contrast, a huge discrepancy was registered about the potential impact of DDR defects on patients’ outcome after targeted radionuclide therapy. As a matter of fact, 3 papers [19,20,24] showed a favorable impact of DDR defects on patients’ outcome, 3 other studies [22,23,25] did not register any significant impact of genetic mutations on final outcome, and 1 paper [21] did not specifically analyze the potential contribution of DDR genes on outcome, but was rather focused on the prevalence of mutations in a selected cohort of subjects with poor response to therapy.

The discrepancy among the aforementioned findings might be explained by several factors. First of all, the retrospective design might have introduced a selection bias. In addition, all the cohorts had a small, sometimes very small (10–13) sample size, and this might have seriously hampered the robustness of statistical analysis [35]. 

It is still debated which is the optimal modality to assess response to radionuclide-based therapies. In this regard, in fact, there is a lack of consensus. In the case of ^223^Ra-therapy, it has been reported that a decline in PSA can be observed only in a minority of subjects, most likely due to the peculiar mechanisms by which this radiopharmaceutical works, since it does not target the androgen receptor but rather bone metabolism. Therefore, ALP measurement has been considered a relevant tool for response evaluation to ^223^Ra-therapy, since it reflects changes in PCa-associated pathologic osteoblastic growth [36]. As far as it concerns the use of the various imaging modalities, bone scans have shown several limitations, especially due to the so-called “bone flare phenomenon”, consisting of a false–positive tracer uptake even in responsive cases, while CT, although useful for detecting new-onset visceral metastases, presents some drawback for the assessment of PCa-skeletal localizations [37]. Similarly, there is still an unmet need for the standardization of response assessment to PSMA-based RLT: in this regard, it has been recently reported that [^68^Ga]Ga-PSMA-11 PET/CT performed 8 weeks after the second cycle of RLT with [^177^Lu]Lu-PSMA-617 is predictive of OS and progressive disease, while PSA is of limited predictive value [38,39]. 

A further consideration has to be made concerning the various radiopharmaceuticals employed for the targeted therapy. It has to be underlined, in fact, that the three studies showing a survival benefit in mCRPC patients with DDR defects were all performed with alpha emitters (^223^Ra-therapy, n = 2; [^225^Ac]Ac-PSMA-617, n = 1), while two of the three papers reporting no clinical benefit in DDR mutated subjects included patients submitted to RLT with [^177^Lu]Lu-PSMA or [^225^Ac]Ac-PSMA-617. This issue might have introduced a further bias, partially explaining the discrepancy among studies. 

**Figure 4:**
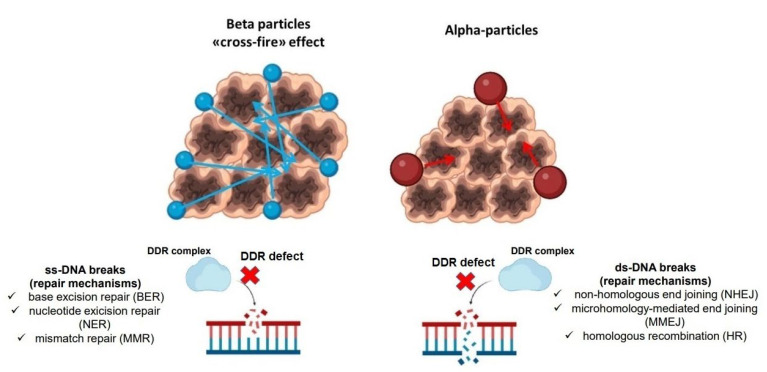
Schematic representation (figure created with biorender.com). Beta-particles (**left side**) have a longer path and interactions in matter, generating the so-called “cross-fire” effect, mainly producing ss-DNA breaks, that can be repaired through specific mechanisms. Alpha-particles (**right side**) have a shorter range in matter with localization to cell nucleus, determining ds-DNA breaks, whose mechanisms of repair are reported. DDR defects can inhibit the complex pathway involved in ss-DNA or ds-DNA-breaks’ repair.

It is worth mentioning, in fact, that radiation-induced damage strictly depends on the energy and type of involved particles. Beta-emitters, such as ^177^Lu or yttrium-90 (^90^Y), have been widely used in clinical practice [40,41,42]: their anti-tumor effects, in spite of particles’ low linear energy transfer (LET), rely on their relatively long range in matter (up to about 11 mm) leading to so-called cross-fire effect and on the indirect damage via reactive oxygen species (ROS); conversely, beta-emitters induce single-strand (ss) DNA breaks, that are relatively easy to be repaired by tumor cells [43,44]. On the contrary, alpha-particles are characterized by a high LET and a shorter range than beta-particles; therefore, their anti-tumor effects require internalization and localization to cell nucleus and, most of all, they determine ds-DNA breaks, independently from cell oxygenation status, difficult to be repaired, and leading to DNA cross-linking and complex chromosomal rearrangement [45,46]. In addition, it has to be underlined that cells employ distinct mechanisms of DDR in relation to the different types of damage (ss-DNA or ds-DNA) [47], as shown in Figure 4. In this perspective, since three studies carried out exclusively with alpha emitters were concordant in registering a survival benefit, it might be reasonable to hypothesize that DDR alterations might have a contributory role in this specific clinical setting.

Finally, all the patients included in the selected papers had been heavily pre-treated with various therapeutic regimens before radionuclide targeted therapy. The optimal sequence of the various available therapies for mCRPC has still to be defined yet [48], however it cannot be excluded that differences in patients’ clinical history before targeted radionuclide therapy might have further contribute to the discrepancy registered among the selected studies. 

## 5. Conclusions

DDR defects are frequently detected in patients affected by advanced PCa submitted to targeted radionuclide therapy. Although biased by the retrospective design and the small sample size, preliminary clinical reports suggest that subjects with DDR mutations might have a trend towards a longer survival when submitted to therapy employing alpha-emitters. Further prospective studies with larger series are needed to better define the role of DDR genes in PCa patients treated with alpha-emitters. 

## Figures and Tables

**Table 1:** Summary of the main findings reported in the selected manuscripts.

Source	Year/Location	Study Design	Sample Size	Demographic Data (Median)	Radiopharmaceutical	Most Common Mutations	Response	Outcome	DDR Impact (Y/N)	Comment
Isaacsson et al. [19]	2019/USA	Retrospective	28 (10 HR+, 35.7%)	Age: 66 yr, Baseline PSA: 77.1 ng/mL, Baseline ALP: 130 U/L	^223^Ra-therapy	Not specified	ALP response within 12 weeks: 64%, with a significantly higher response rate in HR+ patients	More favorable outcome was registered in HR+ than in HR- subjects (OS 36.9 vs. 19.0 mo)	Y	PCa patients harboring mutations in HR genes showed a higher response rate and more prolonged survival after ^223^Ra-therapy
van der Doelen et al. [20]	2020/ The Netherlands + USA	2-centre retrospective study	93 (28 DDR+, 30.1%)	Age: 68 yr, Baseline PSA: 59.0 ng/mL, Baseline ALP: 124 U/L	^223^Ra-therapy	ATM, BRCA2, CDK-12	PSA response: 29.4% in DDR+ vs. 34.6% in DDR-ALP response: 33.0% in DDR+ versus −35.0% in DDR-	OS of the overall cohort: 21 mo DDR+ pts had longer OS than DDR- (median 36.3 vs. 17.0 mo)	Y	Patients harboring DDR alterations had a more favorable outcome after ^223^Ra-therapy. Time to ALP progression (TAP) and time to subsequent treatment (TST) resulted also longer in DDR+ patients than in DDR- ones
Kratochwil et al. [21]	2020/ Germany	Retrospective	10 (7 DDR+, 70%)	Age: not available, Baseline PSA: 481 ng/mL	[^225^Ac]Ac-PSMA-617	TP53, CHEk2, ATM	The study was carried out in non-responders	Not specified	Not assessed	Patients resistant to radioligand therapy with [^225^Ac]Ac-PSMA-617 often harbor mutations in DDR genes
Privé et al. [22]	2021/ The Netherlands	Observational cohort	40 (17 DDR+, 42.5%)	Age: 61 yr, Baseline PSA: not available	[^177^Lu/^225^Ac]-PSMA-617 or PSMA-I&T	BRCA 1/2	No significant differences in PSA response (59% in DDR+ vs. 65% in DDR-)	No OS difference among DDR + patients vs. DDR- patients (median OS 11.1 vs. 10.7 mo)	N	DDR defects did not show any significant impact on mCRPC patients’ response to PSMA-targeted therapy with beta or alpha emitters
Liu et al. [23]	2022/ USA	Two-center retrospective study	127 (127 DDR+)	Age: 61 yr, Baseline PSA: 21 ng/mL, Baseline ALP: 123 U/L	^223^Ra-therapy	TP53, BRCA 1/2, PTEN	PSA response (entire cohort): 22.6%ALP response (entire cohort): 69.8%	TMPRSS2-ERG mutation was associated with a lower OS (15.4 mo), while RB deletion with a shorter PFS (6 mo).	N	DDR mutations did not represent a predictive factor on response to ^223^Ra-therapy, although certain mutations resulted associated with a trend towards a worse prognosis
van der Doelen et al. [24]	2022/ The Netherlans + Germany	Observational cohort	13 (2 DDR+, 15.3%)	Age: 71 yr, Baseline PSA: 878 ng/mL, Baseline ALP: 356 U/L	[^225^Ac]Ac-PSMA-617	BRCA1	PSA response (entire cohort): 69%, ALP response: 62%, RECIST response: 50%, PERCIST response: 86%	OS (entire cohort): 8.5 mo,	Y	Patients harboring DDR defects present a trend toward a longer OS after therapy than those without mutations
Satapathy et al. [25]	2022/ india	Retrospective	15 (10 DDR+, 66.6%)	Age: 66 yr, Baseline PSA: 87 ng/mL	[^177^Lu]Lu-PSMA-617 (n = 3)	ATM, BRCA2, TP53	PSA response (entire cohort): 26.7% RECIST response: 12.5%	PFS (entire cohort): 3 mo, OS (entire cohort): 6 mo	N	DDR defects did not impact either on final outcome or theapy-response of mCRPC patients submitted to RLT

Abbreviations: DDR—DNA damage repair; HR—homologous recombination; yr—years; ALP—alkaline phosphatase; PSA—prostate specific antigen; PSMA —prostate specific membrane antigen; OS—overall survival; PFS—progression free survival; mo—months; Y—yes; N—no.

## Data Availability

Not applicable.
